# Vancomycin-resistant *Enterococcus* infections in a hospital in Salvador, Bahia: a descriptive study, 2021-2023

**DOI:** 10.1590/S2237-96222024v34e20240135.en

**Published:** 2025-04-28

**Authors:** Verônica França Rocha, Matheus Sales Barbosa, Euclimeire da Silva Neves, Valmira de Jesus Santos, Renata Ferreira da Silva Rego, Tiago Lôbo Pessoa, Marcelo Telles Ribeiro, Jessica Lais Almeida dos Santos, Felicidade Mota Pereira, Joice Neves Reis

**Affiliations:** 1 Secretaria de Saúde do Estado da Bahia, Instituto Couto Maia, Salvador, BA, Brazil; 2 Universidade Federal da Bahia, Faculdade de Farmácia, Salvador, BA, Brazil; 3 Secretaria de Saúde do Estado da Bahia, Laboratório Central de Saúde Pública do estado da Bahia, Salvador, BA, Brazil; 4 Fundação Oswaldo Cruz, Instituto Gonçalo Moniz, Salvador, BA, Brazil

**Keywords:** Enterococcus faecalis, Enterococcus faecium, Vancomycin Resistance, Cross Infection, Disease Outbreaks, Enterococcus faecalis, Enterococcus faecium, Resistencia a la Vancomicina, Infección Hospitalaria, Brotes de Enfermedades

## Abstract

**Objective:**

To address the occurrence of an outbreak of vancomycin-resistant Enterococcus in inpatient units intended for COVID-19 patients in a hospital in Salvador, Brazil, specialized in infectious diseases, which underwent several adaptations with effect from March 2020.

**Methods:**

This is a descriptive study of patients with positive culture results for vancomycin-resistant Enterococcus between January 2021 and December 2023. The vanA/vanB genotype was defined by real-time polymerase chain reaction, and the clonal profile, by pulsed-field gel electrophoresis. Descriptive analyses were performed and presented as proportions.

**Results:**

Fifteen Enterococcus spp. infected/colonized patients were identified, of which 7 were E. faecalis, 7 were *E. faecium* and 1 was E. gallinarum. Two clonal profiles were identified: E. faecalis profile A and E. faecium profile C. The vanA genotype was predominant. Possible cross-contamination of disinfected materials and other dirty materials used for bathing and waste disposal was detected in utility/sluice room sinks.

**Conclusion:**

The vancomycin-resistant Enterococcus outbreak in the inpatient units was controlled after implementing a unidirectional flow of disinfection of materials in utility/sluice rooms.

## Resumen

**Objetivo:**

Abordar la ocurrencia de un brote de enterococos resistentes a la vancomicina en unidades de hospitalización destinadas a pacientes con Covid-2019 en un hospital de Salvador, Brasil, especializado en enfermedades infecciosas, que pasó por varias adaptaciones a partir de marzo de 2020.

**Métodos:**

Este es un estudio descriptivo estudio de pacientes con cultivo positivo para enterococos resistentes a vancomicina entre enero de 2021 y diciembre de 2023. El genotipo vanA/vanB se definió mediante reacción en cadena de la polimerasa en tiempo real y el perfil clonal mediante electroforesis de campo pulsado. Se realizaron análisis descriptivos y se presentaron como proporciones.

**Resultados:**

Se identificaron 15 pacientes infectados/colonizados por Enterococcus spp., 7 E. faecalis, 7 E. faecium y 1 E. gallinarum. Se identificaron dos perfiles clonales: el perfil A de E. faecalis y el perfil C de E. faecium. El genotipo vanA fue predominante. En el fregadero de la sala de servicio/purga se detectó posible contaminación cruzada de materiales desinfectados y otros materiales sucios utilizados para bañarse y eliminar desechos.

**Conclusión:**

El brote de enterococos resistentes a vancomicina en unidades de hospitalización fue controlado luego de implementar un flujo unidireccional de desinfección de materiales en la sala de servicio/purga.

## Introduction

Coronavirus 2, responsible for severe acute respiratory syndrome, was first detected in December 2019. Its subsequent evolution into a pandemic subjected health systems in all countries to unprecedented challenges. There was an increase in demand from patients presenting respiratory symptoms related to coronavirus disease 2019 (COVID-19). This led to a high need to increase the number of intensive care beds (1). Management of healthcare-related infections became more complex due to the overload on resources allocated to infection prevention and control measures (2).

Enterococcus spp. are gram-positive cocci that make up the gut microbiota of humans. These bacteria have gained increased attention due to their ability to cause serious infections in critically ill patients, especially when resistant to vancomycin, which can cause urinary tract infection, bloodstream infection and endocarditis (3). Although 61 species of Enterococcus have been identified, E. faecalis and E. faecium are mainly responsible for infection in humans (4). Vancomycin-resistant Enterococcus infections represent a significant challenge in some hospital settings, such as among critically ill patients with COVID-19. 

Some of the risk factors for vancomycin-resistant Enterococcus colonization include: use of antibiotics during hospitalization, surgery and dialysis (5). Severe infections caused by this pathogen are often difficult to treat, requiring combined therapy and management of related adverse effects (6). It has been indicated, through meta-analysis, that infection caused by vancomycin-resistant Enterococcus, regardless of the species, is associated with higher mortality when compared with infections caused by sensitive strains, relative risk (RR) 1.46; 95% interval confidence (95%CI 1.17; 1.82) (7).

Between 2011 and 2014, in the United States, E. faecalis and E. faecium emerged as pathogens responsible for up to 7.4% and 3.7% of all healthcare-associated infections (8). During the COVID-19 crisis, outbreaks of vancomycin-resistant Enterococcus occurred in intensive care units (ICU), where this pathogen was identified in environmental samples. This reinforced the importance of maintaining routine hospital infection control measures even in complex situations such as that (9,10).

During the COVID-19 crisis, a hospital specializing in infectious diseases, located in Salvador, Brazil, underwent several structural and flow modifications to expand its service capacity. During this period, the hospital infection control service identified an increase in cases of infections caused by Enterococcus spp. and the emergence of vancomycin-resistant isolates. This study addressed the occurrence of an outbreak of vancomycin-resistant Enterococcus in inpatient units for patients with COVID-19, with emphasis on adaptations made by the hospital during this challenging period.

## Methods

### Study design

This is a descriptive study conducted in the ICU and wards of the *Instituto Couto Maia*, an infectious diseases hospital located in Salvador.

### Context

The *Instituto Couto Maia* opened its new premises in June 2018 and, as of December 2020, no cases of vancomycin-resistant Enterococcus infection or colonization had been identified.

With the start of the COVID-19 pandemic on March 17, 2020, the Institute adapted to operate exclusively providing care for patients with COVID-19, expanding its capacity from 126 to 162 beds, including 80 ICU beds. Of these 80 ICU beds, 50 were adapted from infirmary beds on the first floor, 20 existing adult beds on the ground floor as well as a further 10 pediatric beds.

### Participants

The research was carried out on patients over 18 years old, admitted to the *Instituto Couto Maia*, between January 2021 and December 2023, with positive culture results for vancomycin-resistant Enterococcus. Patients whose cultures were not available for confirming vancomycin resistance were excluded.

### Variables

The variables analyzed included clinical and demographic information, in addition to the antimicrobial sensitivity profile, defined according to the criteria of the Brazilian Antimicrobial Sensitivity Testing Committee. The infection criteria used were those established by the National Health Surveillance Agency (*Agência Nacional de Vigilância Sanitária*) (11).

### Data sources and measurement

The data were obtained through analysis of records contained in the patients’ medical records. Enterococcus spp. isolates were initially identified and tested for antimicrobial sensitivity in the hospital’s microbiology laboratory, using the Vitek2 system. Strains of vancomycin-resistant Enterococcus, identified in surveillance swabs, were isolated on chromogenic agar – CHROMagar VRE, Plastlabor. The isolates were stored and subsequently sent to the microbiology laboratory of the *Universidade Federal da Bahia* Faculty of Pharmacy to confirm identification of the strain and determine the minimum inhibitory concentration of vancomycin by the agar diffusion method with concentration gradient strips, Etest, BioMérieux, France.

Detection of vanA and vanB genes was performed through real-time polymerase chain reaction using TaqMan probes. Each reaction contained 11.5 μL of GoTaq Probe qPCR Master Mix 2x, PROMEGA, 0.5 μL of sense oligonucleotide (10 μM), 0.5 μL of antisense oligonucleotide (10 μM), 0.5 μL of probe (10 μM) and 8.5 μL of DNAse-free water, and 2 μL of deoxyribonucleic acid was added. The reaction conditions were initial denaturation at 95°C for 2 min followed by 35 to 40 cycles of 95°C for 15s and 60°C for 60s (12). The oligonucleotides and probes used were: VAN-A-F-5’-ATCAACCATGTTGATGTAGC-3’, VAN-A-R-5’-AAGGGATACCGGACAATTCA-3’ and probe-VanA-5’-FAM-TCCATCTTC/ZEN/ACCTGACTTGCCA-3IABKFQ-3; VAN-B-F-5’-ACCCTGTCTTTGTGAAG-3’, VAN-B-R-5’-GAAATCGCTTGCTCAAT-3’ and probe-VanB-HEX-TCCATCATA/ZEN/TTGTCCTGCTGCTTCTAT-3IABKFQ-3’.

The clonal profile of the isolates was determined by the pulsed-field gel electrophoresis method, using digestion of chromosomal DNA with SmaI enzyme. The restriction fragments were separated by agarose gel electrophoresis in the CHEF-DR III apparatus, BioRad, Hercules, CA. The running parameters were: 200 V (6 V/cm); temperature, 13°C; initial switching time 5’ and final time 60’ for 23h. After completion of electrophoresis, the agarose gel was stained in an ethidium bromide solution and visualized on the L-PIX EX, Loccus Biotecnologia, Brazil. The banding pattern was interpreted by visual inspection and analyzed by Gel Compar, Applied Maths NV, Sint-Martens Latem, Belgium. The Unweighted Pair Group Method with Arithmetic Mean was used with 0.75% optimization and 1.00% tolerance. Isolates were classified as identical if they shared the same band profile and as different when they differed by seven or more bands. (13). 

### Study size

This was a descriptive study of 15 cases of patients colonized or infected by vancomycin-resistant Enterococcus at *Instituto Couto Maia* between January 2021 and March 2023.

### Statistical methods

Descriptive statistics were used to describe and characterize the study population in terms of sociodemographic and clinical characteristics. Absolute frequencies were reported to explain categorical variables and medians for continuous measures.

## Results

**Figure 1:**
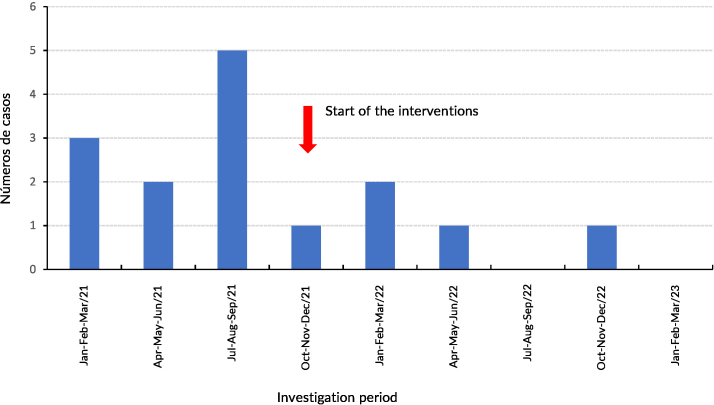
Distribution of vancomycin-resistant Enterococcus infection or colonization cases at the *Instituto Couto Maia*. Salvador, 2021-2023 (n=15)

The first case of vancomycin-resistant Enterococcus was detected on January 13, 2021, in a patient with a bloodstream infection. Between January 2021 and December 2023, 19 patients colonized or infected with vancomycin-resistant Enterococcus were identified. Four cases were excluded due to non-confirmation of resistance. Fifteen patients were included in the study, with a balanced distribution according to gender, 8 of them being male. The patients’ ages ranged from 26 to 81 years, with a median of 49 years. The most frequent comorbidities were COVID-19 infection in 7 cases and human immunodeficiency virus infection in 4 cases (Table 1 and Figure 1). None of the patients underwent surgery during hospitalization.

**Table 1:** Clinical and demographic characteristics of patients with vancomycin-resistant *Enterococcus* infection or colonization. Salvador, 2021-2023 (n=15)

Characteristics	Number
Sex	
Female	7
Male	8
**Age group** (years)	
<30	1
31-45	5
46-60	2
61-75	6
>75	1
**Clinical aspects**	
Number of colonized cases	11
Number of infected cases	4
Central venous catheter-related infection	2
Urinary tract infection	2
Infected deaths	2
Comorbidities/coinfecction	
Hypertension	5
Diabetes	2
Human immunodeficiency virus	4
Nephropathy	2
COVID-19	7
**Use of invasive devices**	
Mechanical ventilation	11
Central venous access/dialysis	13
Indwelling bladder catheter	13
Total	15

Eleven of the 15 patients were identified as being colonized with Vancomycin-resistant Enterococcus, and 4 met criteria for infection. Two of the infected patients died. Average length of hospital stay until the collection of the first culture identifying the pathogen was 41.8 days (ranging from 6 to 104 days). Twelve of the 15 cases were colonized or infected in ICU. Antimicrobial use associated with vancomycin resistance was observed in 9 patients receiving ceftriaxone. One third (5/15) of patients used vancomycin or teicoplanin. Five patients did not use ceftriaxone or glycopeptides. Patients with identification of vancomycin-resistant Enterococcus outside the ICU had been admitted to wards that shared the same corridor and utility room (sluice) with the ICU.

The vancomycin-resistant Enterococcus species were: 7 E. faecalis, 7 *E. faecium* and 1 E. gallinarium. Two isolates were identified in blood cultures; 4, in urine culture; and 9, in rectal swabs. Twelve isolates were sensitive to ampicillin (all 7 E. faecalis isolates being sensitive) and all were sensitive to linezolid and tigecycline (Table 2). The majority (11/15) of isolates were defined as colonization. Among the 4 cases that met the infection criteria, 2 were primary bloodstream infections related to the use of a central venous catheter and 2 were urinary tract infections (Table 1).

**Table 2:** Microbiological profile of Enterococcus spp. isolates regarding sensitivity (S) or resistance (R) to antimicrobials and vancomycin resistance genotype. Salvador, 2021-2023 (n=15)

Case	Species	Source	Date	Teicoplanin	Ampicillin	Gentamicin	Streptomycin	Tigecycline	Linezolid	Vancomycin	Genotype
257	E. faecalis	Blood	20/1/2021	R	S	R	S	S	S	R	VanA
304	E. faecium	Urine	24/2/2021	R	R	S	S	S	S	R	VanA
332	E. faecium	Urine	27/3/2021	R	S	S	S	S	S	R	VanA
412	E. faecalis	Swab	10/9/2021	R	S	R	S	S	S	R	VanA
414	E. faecalis	Swab	3/9/2021	R	S	R	S	S	S	R	VanA
632	E. faecium	Swab	27/4/2022	R	R	S	R	S	S	R	VanA
681	E. gallinarum	Blood	5/5/2022	S	S	R	R	S	S	R	UD^a^
696	E. faecalis	Swab	24/9/2021	R	S	R	S	S	S	R	VanA
697	E. faecalis	Swab	24/9/2021	R	S	R	S	S	S	R	VanA
698	E. faecalis	Swab	20/9/2021	R	S	R	S	S	S	R	VanA
699	E. faecalis	Swab	9/3/2022	R	S	R	S	S	S	R	VanA
704	E. faecium	Urine	21/12/2022	S	R	S	R	S	S	R	UD^a^
705	E. faecium	Urine	7/5/2021	S	R	S	S	S	S	R	UD^a^
706	E. faecium	Swab	9/12/2021	UD^a^	UD^a^	UD^a^	UD^a^	UD^a^	UD^a^	R	UD^a^
707	E. faecium	Swab	9/12/2021	UD^a^	UD^a^	UD^a^	UD^a^	UD^a^	UD^a^	R	UD^a^

^a^UD: undetermined.

**Figure 2:**
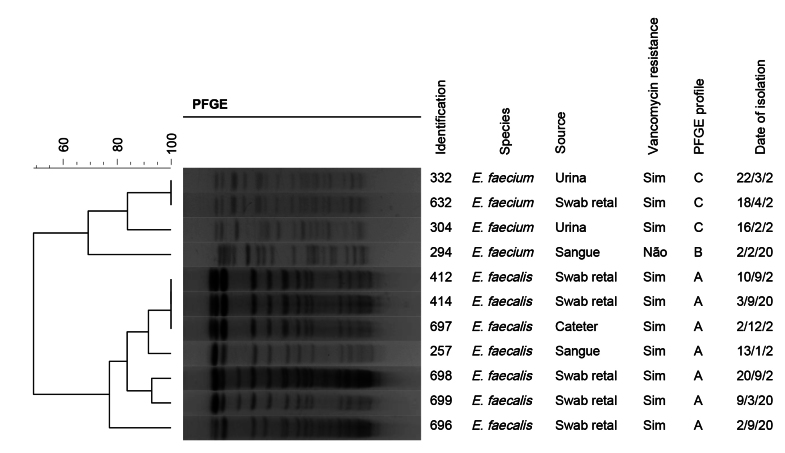
Similarity profile and pulsed-field gel electrophoresis (PFGE) of vancomycin-resistant Enterococcus strains identified at the *Instituto Couto Maia*. Salvador, 2021-2023 (n=11)

Due to problems with storing and sending samples during the COVID-19 crisis, not all isolates were adequately preserved for subsequent analysis of minimum inhibitory concentration, determination of the vanA and vanB genes, and pulsed-field gel electrophoresis. The minimum inhibitory concentration for vancomycin was assessed in 11 of the 15 cases, all of which were higher than 256 µg/mL, with the exception of one E. gallinarum, which presented 8 µg/mL. The vanA gene was identified in all 10 isolates evaluated. The pulsed-field gel electrophoresis profile of the 10 isolates revealed 2 clonal profiles, 7 being profile A and 3 being profile C. Profile A corresponded to E. faecalis isolates, while profile C corresponded to E. faecium. Isolate 294, an E. faecium sensitive to vancomycin, obtained from a patient also admitted to the ICU during the same period, was used as a control and belonged to a different profile, namely profile B (Figure 2).

Clonal isolates, with the exception of one profile A isolate, came from patients admitted to the adapted ICU on the first floor, which had 50 beds and which, before the COVID-19 pandemic, only housed wards. The nursing teams in these adapted ICU used only 2 utility rooms (sluice) for all 50 beds, shared with the team responsible for another 20 infirmary beds located on the same corridor. Clonal profile C was identified exclusively in ICU 3, belonging to the ICU complex on the first floor. Clonal profile A was detected in isolates collected between January 2021 and March 2022. Clonal profile C was identified between February 2021 and April 2022.

As the majority of cases were detected in patients admitted to the adapted ICU on the first floor, the hospital infection control service carried out a technical visit in these sectors. During the inspection, benches and small sinks were observed where utensils used for bathing patients (such as stainless steel jugs and basins) and for disposing of waste (such as stainless steel urine bottles and bedpans) were washed, dried, disinfected and stored on the same surface, without adequate hygiene between stages. These utensils were later stored in cabinets for reuse in the units.

The outbreak control investigation was conducted by the hospital infection control service team, made up of 4 professionals: 2 nurses and 2 infectious disease specialists. The team was responsible for monitoring, managing and internally communicating the outbreak. During this period, patients with confirmed vancomycin-resistant Enterococcus colonization or infection were subjected to contact precautions, isolated in individual rooms. Rectal swabs were collected from all contacts. All patients in the ICU where new cases were detected underwent weekly rectal swabs to test for vancomycin-resistant Enterococcus. This active surveillance was maintained until the sector went 3 weeks without new cases being detected.

With effect from November 2021, progressive interventions were carried out in the care unit sluice rooms, in order to segregate material disinfection stages. Dirty utensils, such as bedpans and basins, continued to be left on the sink, but, after washing, they began to be placed on a hollow stainless steel shelf, installed above the sink, for drying, so as to avoid cross-contamination. A stainless steel table was installed in the sluice room for exclusive disinfection of washed and dried utensils. These utensils were then stored in plastic bags, kept in exclusive cabinets.

Restructuring of the sluice rooms in all care units began in November 2021 and was completed in May 2022. From June 2022 to December 2023, only 1 case was recorded. During the entire period of the outbreak and after its control, the disinfectant used in the institution was fifth generation quaternary ammonia.

## Discussion

This study describes an outbreak of 15 cases of vancomycin-resistant Enterococcus infection/colonization, which occurred in a hospital in Salvador that had undergone several structural interventions due to the COVID-19 pandemic. Of particular note was the adaptation of 50 infirmary beds to ICU beds, which increased the utility/sluice room demand for cleaning utensils. The undersized physical structure and the absence of unidirectional disinfection flow may have facilitated cross-contamination of materials. 

The utilities/sluice room is an environment intended for cleaning, disinfecting and storing materials used in patient care. According to National Health Surveillance Agency Resolution No. 50, dated February 21, 2002, which provides for planning, programming, preparation, evaluation and approval of physical projects for healthcare establishments, whether public or private, the utility/sluice room is described as a support sector for various healthcare sectors (14). Although the Resolution details the size of the utility/sluice room, as well as the infrastructure necessary for its operation, it does not specify the correct unidirectional flow for disinfection of materials, in order to prevent cross-contamination between dirty utensils and others already disinfected. In order to enable unidirectional disinfection flow in the sluice room, a stainless steel shelf had to be installed, this being an item not specified by the Resolution, but which was essential to ensure that the materials were not dried on the sinks, where the team also placed dirty material after patient use.

The spatial individualization of each stage of utensil disinfection provided a unidirectional flow of material disinfection and avoided cross-contamination between dirty and clean items, without changing any other flow/process in the unit. The restructuring of all sluice rooms in the hospital’s ICU and wards was followed by interruption in the detection of new cases in all hospital care sectors. Control of cases after the utility/sluice room intervention, combined with the clonal similarity between Enterococcus spp. isolates, suggests a common source of infection acquisition.

To prevent the spread of the pathogen to other sectors of the hospital, interventions were carried out in all sluice rooms, such as those in the ICU on the first floor (50 beds), emergency, pediatrics and the ICU on the ground floor (20 beds). The large outbreak of vancomycin-resistant Enterococcus that occurred in a hospital in Switzerland, with identification of 518 cases over a period of 32 months, was initially unsuccessful in controlling cases by adopting an infection control strategy restricted to units with cases. Only after implementing a multimodal strategy throughout the entire hospital was it possible to control the spread of the ST796 clone, considered highly transmissible. The measures implemented were the creation of separate cohort areas for patients confirmed as having vancomycin-resistant Enterococcus, the performance of weekly surveillance rectal swabs in patients admitted to high-risk units, in addition to screening devices such as computers, ultrasound equipment and surfaces in the nursing sector (15).

In this study, isolate 699, belonging to clonal profile A, was identified in a patient who had never been admitted to the adapted ICU on the first floor. Until the strain was identified, the patient remained only in the ground floor ICU, demonstrating the importance of implementing strategies beyond the sector where cases were identified. The two identified clonal profiles, A and C, were detected for one year, which could be explained by weaknesses in environmental hygiene associated with continuing cross-contamination of materials. It is important to highlight the high turnover of health professionals in this period of crisis caused by COVID-19, in addition to the rapid hiring of people with little experience due to the high demand for intensive beds, which may have contributed to the weaknesses of the process. 

Although some studies have questioned the need to implement contact precautions for patients colonized and infected with vancomycin-resistant Enterococcus, all cases in this outbreak were subjected to contact precautions (16,17). The remaining patients who had been in contact with the cases were subjected to contact precautions, but only underwent surveillance swabs. A study showed that in 12 hospitals discontinuing contact precautions for patients with vancomycin-resistant Enterococcus or oxacillin-resistant Staphylococcus aureus did not increase infection rates, even in hospitals with a small proportion of private rooms (16). All of these hospitals had low rates of infections related to these pathogens, in addition to high adherence to hand hygiene (16).

The impact of COVID-19 on the incidence of multidrug-resistant bacteria and their respective infections may vary in each institution. The following was identified in a university hospital in the city of São Paulo: an increase in infections due to carbapenem-resistant *Acinetobacter baumannii*, carbapenem-resistant Enterobacterales and oxacillin-resistant *Staphylococcus aureus*; and reduction in the incidence of vancomycin-resistant Enterococcus infections (18). Adherence to hospital infection control practices and management of antibiotic use are critical for controlling and preventing outbreaks of vancomycin-resistant Enterococcus. This may explain the differences in the incidence of infections/colonizations between different institutions during that challenging period (9).

More than half of the patients in this study used ceftriaxone, and a third used some type of glycopeptide. Average length of hospital stay was 41.8 days. Use of ceftriaxone and prolonged hospitalization have been previously associated with vancomycin-resistant Enterococcus colonization and infection in an outbreak caused by vanA strains (19). A case-control study that assessed the risk of bacteremia caused by this pathogen in previously colonized patients identified that use of carbapenems and cephalosporins was an independent risk factor for infection, OR 6.67; 95%CI 1.30; 34; p-valor=0.022 and OR 4.32; 95%CI 1.23; 15; p-valor=0.022, respectively (20).

Standing out among the limitations of this study was the failure to perform genome multilocus sequence typing. This could help in understanding the pattern of case dissemination, as some clonal types, such as ST796, spread more quickly (21). Environmental cultures were also not carried out. This could allow for better correlation between patient isolates and the possible location of the origin of the outbreak, as demonstrated in an outbreak in an ICU in Germany. That outbreak showed genetic correlation between clones identified on contaminated surfaces and those isolated from patients (10).

This study may have selection bias, as cases were identified based on surveillance culture results and diagnostic procedures for detecting vancomycin-resistant Enterococcus. According to hospital records, 19 cases with positive cultures occurred during the period. Isolates were available for microbiological and molecular characterization for only 15 cases.

The outbreak of vancomycin-resistant Enterococcus faced in ICU dedicated to COVID-19 patients has highlighted critical vulnerabilities in infection control practices. Interventions focused on reviewing and restructuring cleaning and disinfection procedures were decisive in stopping the outbreak. Identification of sluice room sinks as a potential cross-contamination point between dirty and already disinfected materials as well as the subsequent intervention to avoid this contact seem to have contributed to the control of cases. The identification of two clonal profiles among the isolates reinforces the complexity of infection control and the importance of dynamic adaptation to changes in transmission patterns.

This study reinforces the need for strict control of hospital infections, especially during pandemics, by presenting an approach to investigation and control of an outbreak of vancomycin-resistant Enterococcus. Cross-contamination between disinfected and soiled materials in utility/sluice room sinks was identified as a contributing factor. Corrective measures, such as organizing materials in specific locations and ensuring unidirectional disinfection flow, were essential for controlling the outbreak, in accordance with the good practices recommended by National Health Surveillance Agency Resolution RDC 63/2011, which provides guidance on the adoption of cleaning and disinfection procedures in critical areas. We suggest that a manual be developed on good practices for hospital support areas, such as sluice rooms, as also recommended by Resolution RDC 50/2002, in order to prevent outbreaks and control infections related to healthcare.
