# Supplementary material for: Vancomycin-resistant Enterococcus infections in a hospital in Salvador, Bahia: a descriptive study, 2021-2023
Source: Epidemiol Serv Saude. 2025 Apr 28;34:e20240135. doi: 10.1590/S2237-96222024v34e20240135.en (PMC12039790; doi:10.1590/S2237-96222024v34e20240135.en)
Supplement: Translation

# Translation: Infecções por Enterococcus resistentes à vancomicina em um hospital de Salvador, Bahia: estudo descritivo, 2021-2023

**DOI:** 10.1590/S2237-96222024v34e20240135.pt

**Authors:** Verônica França Rocha, Matheus Sales Barbosa, Euclimeire da Silva Neves, Valmira de Jesus Santos, Renata Ferreira da Silva Rego, Tiago Lôbo Pessoa, Marcelo Telles Ribeiro, Jessica Lais Almeida dos Santos, Felicidade Mota Pereira, Joice Neves Reis

**Affiliations:** 1 Secretaria de Saúde do Estado da Bahia, Instituto Couto Maia, Salvador, BA, Brasil; 2 Universidade Federal da Bahia, Faculdade de Farmácia, Salvador, BA, Brasil; 3 Secretaria de Saúde do Estado da Bahia, Laboratório Central de Saúde Pública do estado da Bahia, Salvador, BA, Brasil; 4 Fundação Oswaldo Cruz, Instituto Gonçalo Moniz, Salvador, BA, Brasil

**Keywords:** Enterococcus faecalis, Enterococcus faecium, Resistência à Vancomicina, Infecção Hospitalar, Surtos de Doenças

## Abstract

**Objetivo:**

Abordar a ocorrência de surto de Enterococcus resistentes à vancomicina nas unidades de internação destinadas a pacientes com covid-2019 de um hospital de Salvador, especializado em infectologia, que foi submetido a diversas adaptações a partir de março de 2020.

**Métodos:**

Tratou-se de estudo descritivo de pacientes com cultura positiva para *Enterococcus* resistentes à vancomicina entre janeiro de 2021 e dezembro de 2023. O genótipo vanA/vanB foi definido por reação em cadeia da polimerase em tempo-real, e o perfil clonal, pela eletroforese em campo pulsado. Análises descritivas foram realizadas e apresentadas como proporções.

**Resultados:**

Identificaram-se 15 pacientes infectados/colonizados por *Enterococcus* spp., sendo 7 E. faecalis, 7 *E. faecium* e 1 E. gallinarum. Dois perfis clonais foram identificados: perfil A de E. faecalis e perfil C de E. faecium. O genótipo *vanA* foi predominante. Detectou-se possível contaminação cruzada de materiais desinfetados e outros com sujidades, utilizados para banho e descarte das dejeções, na pia da sala de utilidades/expurgo.

**Conclusão:**

O surto de *Enterococcus* resistentes à vancomicina nas unidades assistenciais foi controlado após implantação de fluxo unidirecional de desinfecção de materiais na sala de utilidades/expurgo.

## Introdução

A primeira detecção do coronavírus 2, responsável pela síndrome respiratória aguda grave, ocorreu em dezembro de 2019. A subsequente evolução para uma pandemia submeteu os sistemas de saúde de todos os países a desafios sem precedentes. Houve aumento da demanda de pacientes que apresentavam sintomas respiratórios relacionados à doença por coronavírus 2019 (covid-19). Isso ocasionou a elevada necessidade de aumento do número de leitos de terapia intensiva (1). A gestão de infecções relacionadas à assistência à saúde tornou-se mais complexa com a sobrecarga dos recursos destinados às medidas de prevenção e controle das infecções (2).

Enterococcus spp. são cocos gram-positivos que compõem a microbiota gastrointestinal dos seres humanos. Essas bactérias ganharam destaque devido à sua capacidade de causar infecções graves em pacientes críticos, especialmente quando resistentes à vancomicina, podendo causar infecção do trato urinário, infecção de corrente sanguínea e endocardite (3). Apesar de 61 espécies de enterococos terem sido identificadas, E. faecalis e E. faecium são os principais responsáveis pela infecção em humanos (4). As infecções por *Enterococcus* resistentes à vancomicina representam desafio significativo em alguns contextos hospitalares, tais como no paciente crítico com covid-19. 

Alguns dos fatores de risco para colonização por *Enterococcus* resistentes à vancomicina incluem: uso de antibióticos durante a internação, cirurgia e diálise (5). Infecções graves por esse patógeno costumam ser de difícil tratamento, exigindo terapia combinada e manejo dos efeitos adversos relacionados (6). Indicou-se, por meio de meta-análise, que a infecção por *Enterococcus* resistentes à vancomicina, independentemente da espécie, está associada à mortalidade mais elevada quando comparada com infecções por cepas sensí­veis, risco relativo (RR) 1,46; intervalo de confiança de 95% (IC95% 1,17; 1,82) (7).

Entre 2011 e 2014, nos Estados Unidos, E. faecalis e E. faecium surgiram como patógenos responsáveis por até 7,4% e 3,7% de todas as infecções relacionadas à assistência à saúde (8). Durante a crise causada pela covid-19, surtos de *Enterococcus* resistentes à vancomicina ocorreram em unidades de terapia intensiva (UTI), onde foi identificado esse patógeno em amostras ambientais. Isso reforçou a importância da manutenção das medidas de rotina de controle de infecção hospitalar mesmo em situações complexas como essa (9,10).

No período de crise da covid-19, um hospital especializado em doenças infecciosas, localizado em Salvador, passou por diversas modificações estruturais e de fluxos para ampliar a capacidade de atendimento. Nesse período, o serviço de controle de infecção hospitalar identificou o aumento de casos de infecções por Enterococcus spp. e o surgimento de isolados resistentes à vancomicina. Este estudo abordou a ocorrência de um surto de *Enterococcus* resistentes à vancomicina nas unidades de internação para pacientes com covid-19, destacando as adaptações realizadas pelo hospital durante esse período desafiador.

## Métodos

### Delineamento do estudo

Trata-se de estudo descritivo conduzido nas UTI e enfermarias do Instituto Couto Maia, hospital de doenças infecciosas localizado em Salvador.

### Contexto

O Instituto Couto Maia inaugurou a nova sede em junho de 2018 e, até dezembro de 2020, nenhum caso de infecção ou colonização por Enterococcus resistentes à vancomicina havia sido identificado.

Com o início da pandemia da covid-19 em 17 de março de 2020, o instituto adaptou-se para funcionar exclusivamente no atendimento a pacientes com covid-19, expandindo sua capacidade de 126 para 162 leitos, incluindo 80 leitos de UTI. Destes 80 leitos de UTI, 50 foram adaptados a partir de leitos de enfermaria no primeiro andar, 20 leitos de adulto já existentes no térreo e outros 10 de pediatria. 

### Participantes

A pesquisa foi realizada em pacientes maiores de 18 anos, internados no Instituto Couto Maia, entre janeiro de 2021 e dezembro de 2023, com culturas positivas para Enterococcus resistente à vancomicina. Pacientes cujas culturas não estavam disponíveis para confirmação de resistência à vancomicina foram excluídos.

### Variáveis

As variáveis analisadas incluíram informações clínicas e demográficas, além do perfil de sensibilidade antimicrobiana, definidos conforme os critérios do Comitê Brasileiro de Testes de Sensibilidade aos Antimicrobianos. Os critérios de infecção utilizados foram aqueles estabelecidos pela Agência de Vigilância Sanitária (11).

### Fontes de dados e mensuração

Os dados foram obtidos por meio da análise dos registros contidos nos prontuários dos pacientes. Os isolados de Enterococcus spp. foram inicialmente identificados e testados quanto à sensibilidade antimicrobiana no laboratório de microbiologia do hospital, utilizando o sistema Vitek2. Cepas de *Enterococcus* resistentes à vancomicina, identificadas em swab de vigilância, foram isoladas em ágar cromogênico – CHROMagar VRE, Plastlabor. Os isolados foram armazenados e posteriormente enviados ao laboratório de microbiologia da Faculdade de Farmácia da Universidade Federal da Bahia para confirmação da identificação da cepa e determinação da concentração inibitória mínima da vancomicina pelo método de difusão em ágar com fitas de gradiente de concentração, Etest, BioMérieux, França.

A detecção dos genes vanA e vanB foi realizada por reação em cadeia da polimerase em tempo-real utilizando sondas TaqMan. Cada reação continha 11,5 μL de GoTaq Probe qPCR Master Mix 2x, PROMEGA, 0,5 μL de oligonucletídeo senso (10 μM), 0,5 μL de oligonucletídeo antisenso (10 μM), 0,5 μL de sonda (10 μM) e 8,5 μL de água livre de DNA se, e adicionou-se 2 μL de ácido desoxirribonucleico. As condições da reação foram uma desnaturação inicial a 95°C por 2 min seguida de 35 a 40 ciclos de 95°C por 15s e 60°C por 60s (12). Os oligonucleotídeos e sondas utilizados foram: VAN-A-F-5’-ATCAACCATGTTGATGTAGC-3’, VAN-A-R-5’-AAGGGATACCGGACAATTCA-3’ e sonda-VanA-5’-FAM-TCCATCTTC/ZEN/ACCTGACTTGCCA-3IABKFQ-3; VAN-B-F-5’-ACCCTGTCTTTGTGAAG-3’, VAN-B-R-5’-GAAATCGCTTGCTCAAT-3’ e sonda-VanB-HEX-TCCATCATA/ZEN/TTGTCCTGCTGCTTCTAT-3IABKFQ-3’.

O perfil clonal dos isolados foi determinado pelo método de eletroforese em campo pulsado, empregando a digestão do DNA cromossômico com a enzima SmaI. Os fragmentos de restrição foram separados por eletroforese em gel de agarose no aparelho CHEF-DR III, BioRad, Hercules, CA. Os parâmetros de corrida foram: 200 V (6 V/cm); temperatura, 13°C; tempo de comutação inicial 5’ e final 60’ por 23h. Após a conclusão da eletroforese, o gel de agarose foi corado em uma solução de brometo de etídio e visualizado no L-PIX EX, Loccus Biotecnologia, Brasil. O padrão de bandas foi interpretado por inspeção visual e analisado por Gel Compar, Applied Maths NV, Sint-Martens Latem, Bélgica. Foi utilizado o método Unweighted Pair Group Method with Arithmetic Average com otimização de 0,75% e tolerância de 1,00%. Os isolados foram classificados como idênticos se compartilhassem o mesmo perfil de bandas e como diferentes quando divergiam por sete ou mais bandas (13). 

### Tamanho do estudo

Estudo descritivo de 15 casos de pacientes colonizados ou infectados por *Enterococcus* resistentes à vancomicina no Instituto Couto Maia entre janeiro de 2021 e março de 2023.

### Métodos estatísticos

Estatísticas descritivas foram usadas para descrever e caracterizar a população do estudo em termos de características sociodemográficas e clínicas. Frequências absolutas foram relatadas para explicar variáveis categóricas e mediana para medidas contínuas.

## Resultados

O primeiro caso de Enterococcus resistente à vancomicina foi detectado em 13 de janeiro de 2021, em um paciente com infecção de corrente sanguínea. Entre janeiro de 2021 e dezembro de 2023, foram identificados 19 pacientes colonizados ou infectados por Enterococcus resistentes à vancomicina. Quatro casos foram excluídos devido à não confirmação da resistência. Quinze pacientes foram incluídos no estudo, com distribuição equilibrada por gênero, sendo 8 casos do sexo masculino. A idade dos pacientes variou de 26 a 81 anos, com mediana de 49 anos. As comorbidades mais frequentes foram infecção por covid-19 em 7 casos e infecção pelo vírus da imunodeficiência humana em 4 casos (Tabela 1 e Figura 1). Nenhum dos pacientes foi submetido à cirurgia durante a internação.

**Tabela 1:** Características clínicas e demográficas dos pacientes com infecção ou colonização por Enterococcus resistentes à vancomicina. Salvador, 2021-2023 (n=15)

Características	Número
Sexo	
Feminino	7
Masculino	8
**Faixa etária** (anos)	
<30	1
31-45	5
46-60	2
61-75	6
>75	1
**Aspectos clínicos**	
Número de colonizados	11
Número de infectados	4
Infecção relacionada a cateter venoso central	2
Infecção do trato urinário	2
Óbito dos infectados	2
Comorbidades/coinfecção	
Hipertensão arterial sistêmica	5
Diabetes	2
Vírus da imunodeficiência humana	4
Nefropatia	2
Covid-19	7
**Uso de dispositivos invasivos**	
Ventilação mecânica	11
Acesso venoso central/diálise	13
Sonda vesical de demora	13
Total	**15**

**Figura 1:** Distribuição de casos de infecção ou colonização por Enterococcus resistentes à vancomicina no Instituto Couto Maia. Salvador, 2021-2023 (n=15)

Onze dos 15 pacientes foram identificados como colonizados por *Enterococcus* resistentes à vancomicina, e 4 preencheram os critérios para infecção. Entre os pacientes infectados, 2 evoluíram para óbito. O tempo médio de internação hospitalar até a coleta da primeira cultura com identificação do patógeno foi 41,8 dias (variando de 6 a 104 dias). Doze dos 15 casos foram colonizados ou infectados em UTI. O uso de antimicrobianos associados à resistência à vancomicina foi observado em 9 pacientes que receberam ceftriaxona. Um terço (5/15) dos pacientes utilizaram vancomicina ou teicoplanina. Cinco pacientes não usaram ceftriaxona ou glicopeptídeos. Os pacientes com identificação de Enterococcus resistentes à vancomicina fora das UTI estavam internados em enfermarias que compartilhavam o mesmo corredor e sala de utilidades (expurgo) com as UTI.

As espécies de Enterococcus resistentes à vancomicina foram: 7 E. faecalis, 7 *E. faecium* e 1 E. gallinarium. Dois isolados foram identificados em hemoculturas; 4, em urocultura; e 9, em swab retal. Doze isolados foram sensíveis à ampicilina (sendo todos os 7 isolados de *E. faecalis* sensíveis) e todos sensíveis *à* linezolida e tigeciclina (Tabela 2). A maioria (11/15) dos isolados foi definida como de colonização. Entre os 4 casos que preencheram critério de infecção, 2 foram de infecção primária de corrente sanguínea relacionada ao uso de cateter venoso central e 2 foram infecção do trato urinário (Tabela 1).

**Tabela 2:** Perfil microbiológico dos isolados de *Enterococcus* spp. quanto à sensibilidade (S) ou resistência (R) aos antimicrobianos e genótipo de resistência à vancomicina. Salvador, 2021-2023 (n=15)

Caso	Espécie	Fonte	Data	Teicoplanina	Ampicilina	Gentamicina	Estreptomicina	Tigeciclina	Linezolida	Vancomicina	Genótipo
257	E. faecalis	Sangue	20/1/2021	R	S	R	S	S	S	R	VanA
304	E. faecium	Urina	24/2/2021	R	R	S	S	S	S	R	VanA
332	E. faecium	Urina	27/3/2021	R	S	S	S	S	S	R	VanA
412	E. faecalis	Swab	10/9/2021	R	S	R	S	S	S	R	VanA
414	E. faecalis	Swab	3/9/2021	R	S	R	S	S	S	R	VanA
632	E. faecium	Swab	27/4/2022	R	R	S	R	S	S	R	VanA
681	E. gallinarum	Sangue	5/5/2022	S	S	R	R	S	S	R	ND^a^
696	E. faecalis	Swab	24/9/2021	R	S	R	S	S	S	R	VanA
697	E. faecalis	Swab	24/9/2021	R	S	R	S	S	S	R	VanA
698	E. faecalis	Swab	20/9/2021	R	S	R	S	S	S	R	VanA
699	E. faecalis	Swab	9/3/2022	R	S	R	S	S	S	R	VanA
704	E. faecium	Urina	21/12/2022	S	R	S	R	S	S	R	ND^a^
705	E. faecium	Urina	7/5/2021	S	R	S	S	S	S	R	ND^a^
706	E. faecium	Swab	9/12/2021	ND^a^	ND^a^	ND^a^	ND^a^	ND^a^	ND^a^	R	ND^a^
707	E. faecium	Swab	9/12/2021	ND^a^	ND^a^	ND^a^	ND^a^	ND^a^	ND^a^	R	ND^a^

^a^ND: não determinado.

Devido a problemas de armazenamento e envio de amostras durante a crise de covid-19, nem todos os isolados foram adequadamente preservados para posterior análise da concentração inibitória mínima, determinação dos genes vanA e vanB e eletroforese em campo pulsado. A concentração inibitória mínima para vancomicina foi avaliada em 11 dos 15 casos, sendo todas superiores a 256 µg/mL, com exceção de um E. gallinarum, que apresentou 8 µg/mL. O gene vanA foi identificado em todos os 10 isolados avaliados. O perfil de eletroforese em campo pulsado dos 10 isolados revelou 2 perfis clonais, sendo 7 do perfil A e 3 do perfil C. O perfil A correspondeu a isolados de E. faecalis, enquanto o perfil C foi de E. faecium. O isolado 294, um E. faecium sensível à vancomicina, obtido de um paciente também internado em UTI durante o mesmo período, foi utilizado como controle e pertenceu a um perfil distinto, o perfil B (Figura 2).

**Figura 2:** Perfil de similaridade e eletroforese em gel de campo pulsado (PFGE) das cepas de Enterococcus resistentes à vancomicina identificadas no Instituto Couto Maia. Salvador, 2021-2023 (n=11)

Isolados clonais, com exceção de um isolado do perfil A, foram provenientes de pacientes internados nas UTI adaptadas do primeiro andar, que contavam com 50 leitos e que, antes da pandemia de covid-19, abrigavam apenas enfermarias. As equipes de enfermagem dessas UTI adaptadas utilizavam apenas 2 salas de utilidades (expurgo) para todos os 50 leitos, compartilhadas com a equipe responsável por outros 20 leitos de enfermaria localizados no mesmo corredor. O perfil clonal C foi identificado exclusivamente na UTI 3, pertencente ao complexo de UTI do primeiro andar. O perfil clonal A foi detectado em isolados coletados entre janeiro de 2021 e março de 2022. O perfil clonal C foi identificado entre fevereiro de 2021 e abril de 2022.

Como a maioria dos casos foi detectada em pacientes internados nas UTI adaptadas do primeiro andar, o serviço de controle de infecção hospitalar realizou visita técnica a esses setores. Durante a inspeção, foram observadas bancadas e pias pequenas onde utensílios usados no banho dos pacientes (como jarras e bacias de inox) e na eliminação de dejetos (como papagaios e comadres de inox) eram lavados, secos, desinfetados e armazenados na mesma superfície, sem higienização adequada entre as etapas. Esses utensílios eram posteriormente guardados em armários para reutilização nas unidades.

A investigação do controle do surto foi conduzida pela equipe do serviço de controle de infecção hospitalar, composta por 4 profissionais: 2 enfermeiros e 2 infectologistas. A equipe foi responsável pelo monitoramento, pelo gerenciamento e pela comunicação interna do surto. Durante o período, os pacientes com confirmação de colonização ou infecção por Enterococcus resistentes à vancomicina foram colocados em precaução de contato, isolados em quartos individuais. Realizou-se a coleta de swab retal em todos os contactantes. Todos os pacientes das UTI onde novos casos eram detectados foram submetidos semanalmente a swab retal para pesquisa de Enterococcus resistentes à vancomicina. Essa vigilância ativa foi mantida até que o setor ficasse 3 semanas sem a detecção de novos casos.

A partir de novembro de 2021, intervenções progressivas foram realizadas nas salas de expurgo das unidades assistenciais, para segregar etapas da desinfecção de materiais. Utensílios sujos, como comadres e bacias, continuaram sendo depositados sobre a pia, mas, após a lavagem, passaram a ser colocados em uma prateleira vazada de inox, instalada acima da pia, para secagem, evitando a contaminação cruzada. Uma mesa de inox foi instalada no expurgo para desinfecção exclusiva de utensílios lavados e secos. O armazenamento desses utensílios passou a ser feito em sacolas plásticas, guardadas em armários exclusivos.

A reestruturação das salas de utilidades de todas as unidades assistenciais foi iniciada em novembro de 2021 e finalizada em maio de 2022. De junho de 2022 até dezembro de 2023, foi registrado apenas 1 caso. Durante todo o período do surto e após o seu controle, o desinfetante utilizado na instituição foi o quaternário de amônia de quinta geração.

## Discussão

Este estudo descreve um surto de 15 casos de infecção/colonização por Enterococcus resistente à vancomicina, ocorrido em um hospital de Salvador que passou por diversas intervenções estruturais devido à pandemia de covid-19. Destacou-se a adaptação de 50 leitos de enfermaria para UTI, o que aumentou a demanda pela sala de utilidades/expurgo para higienização de utensílios. A estrutura física subdimensionada e a ausência de fluxo unidirecional de desinfecção podem ter facilitado a contaminação cruzada dos materiais. 

A sala de utilidades/expurgo é um ambiente destinado à limpeza, desinfecção e guarda de materiais utilizados na assistência ao paciente. Conforme a Resolução nº 50, de 21 de fevereiro de 2002, da Agência Nacional de Vigilância Sanitária, que trata do planejamento, programação, elaboração, avaliação e aprovação de projetos físicos de estabelecimentos assistenciais de saúde, público ou privado, a sala de utilidades/expurgo é descrita como setor de apoio para diversos setores assistenciais (14). Embora esse documento detalhe o dimensionamento da sala de utilidades/expurgo, assim como a infraestrutura necessária para o seu funcionamento, não especifica o fluxo correto unidirecional de desinfecção dos materiais, de forma a impedir o cruzamento de utensílios contaminados com aqueles já desinfetados. Para possibilitar o fluxo unidirecional de desinfecção na sala de utilidades, foi necessário instalar uma prateleira de inox, item não especificado pela resolução, mas que foi fundamental para assegurar que os materiais não fossem secos sobre as pias, onde a equipe colocava o material sujo após o uso no paciente.

A individualização espacial de cada etapa da desinfecção dos utensílios proporcionou o fluxo unidirecional da desinfecção dos materiais e evitou o cruzamento de itens sujos e limpos, sem alterar qualquer outro fluxo/processo na unidade. A reestruturação de todas as salas de utilidades das UTI e enfermarias do hospital foi seguida pela interrupção na detecção de novos casos em todos os setores assistenciais do hospital. O controle dos casos após a intervenção na sala de utilidades/expurgo, aliado à similaridade clonal entre os isolados de *Enterococcus* spp., sugere fonte comum de aquisição.

Para evitar a disseminação do patógeno para outros setores do hospital, intervenções foram realizadas em todas as salas de utilidades, como as dos leitos de UTI do primeiro andar (50 leitos), emergência, pediatria e as UTI do térreo (20 leitos). O grande surto de *Enterococcus* resistentes à vancomicina ocorrido em um hospital da Suíça, com a identificação de 518 casos em um período de 32 meses, inicialmente não teve sucesso no controle dos casos ao adotar a estratégia de controle de infecção restrita às unidades com casos. Apenas após a implementação de estratégia multimodal em todo o hospital, foi possível controlar a propagação do clone ST796, considerado altamente transmissível. As medidas implementadas foram a criação de áreas de coorte distintas para pacientes confirmados com *Enterococcus* resistentes à vancomicina, a realização de swab retal de vigilância semanal em pacientes internados em unidades de alto risco, além de rastreamento em dispositivos como computadores e equipamentos de ultrassonografia e em superfícies no setor de enfermagem (15).

Neste estudo, o isolado 699, pertencente ao perfil clonal A, foi identificado em um paciente que nunca havia sido internado nas UTI adaptadas do primeiro andar. Até o momento de identificação da cepa, o paciente permaneceu apenas na UTI do térreo, demonstrando a importância de implementar estratégias além do setor onde houve identificação de casos. Os dois perfis clonais identificados, A e C, foram detectados por um ano, o que poderia ser explicado por fragilidades da higienização do ambiente associadas à manutenção da contaminação cruzada dos materiais. É importante ressaltar a alta rotatividade de profissionais de saúde nesse período de crise causada pela covid-19, além da contratação rápida de pessoas com pouco tempo de experiência devido à alta demanda por leitos intensivos, o que pode ter contribuído com as fragilidades do processo. 

Apesar de alguns estudos questionarem a necessidade de implementar precauções de contato para pacientes colonizados e infectados por Enterococcus resistentes à vancomicina, todos os casos desse surto foram colocados em precaução de contato (16,17). Os demais pacientes contactantes dos casos não foram colocados em precaução de contato, mas apenas submetidos a swab de vigilância. Em 12 hospitais, evidenciou-se que descontinuar a precaução de contato de pacientes com *Enterococcus* resistentes à vancomicina ou *Staphylococcus aureus* resistente à oxacilina não aumentou as taxas de infecções, mesmo em hospitais com pequena proporção de leitos privativos (16). Todos esses hospitais apresentavam baixas taxas de infecções relacionadas a esses patógenos, além de elevada adesão à higienização das mãos (16).

O impacto da covid-19 na incidência de bactérias multirresistentes e suas respectivas infecções pode variar em cada instituição. Em um hospital universitário da cidade de São Paulo, identificaram-se: aumento de infecções por *Acinetobacter baumannii* resistente a carbapenêmicos, *Enterobacterales* resistente a carbapenêmicos e *Staphylococcus aureus* resistente à oxacilina; e redução na incidência de infecções por *Enterococcus* resistentes à vancomicina (18). A adesão às práticas de controle de infecção hospitalar e o gerenciamento do uso de antibióticos são fundamentais para controlar e evitar surtos de *Enterococcus* resistentes à vancomicina. Isso pode explicar as diferenças nas incidências de infecções/colonizações entre as diversas instituições nesse período desafiador (9).

Mais da metade dos pacientes deste estudo fizeram uso de ceftriaxona, e um terço fez uso de algum glicopeptídeo. O tempo médio de internação hospitalar foi 41,8 dias. O uso de ceftriaxona e o tempo prolongado de internação já foram associados à colonização e infecção por *Enterococcus* resistentes à vancomicina em um surto por cepas *vanA* (19). Um estudo caso-controle que avaliou risco de bacteremia por esse patógeno em pacientes previamente colonizados identificou que o uso de carbapenêmicos e cefalosporinas foi fator de risco independente para infecção, OR 6,67; IC95% 1,30; 34; p-valor=0,022 e OR 4,32; 95%IC 1,23; 15; p-valor=0,022, respectivamente (20).

Entre as limitações deste estudo, destacou-se a não realização da tipagem de sequência multilocus do genoma. Esta poderia auxiliar na compreensão do padrão da disseminação dos casos, já que alguns tipos clonais, como o ST796, se propagam mais rapidamente (21). Também não foram realizadas culturas do ambiente. Isso poderia permitir a melhor correlação entre os isolados dos pacientes e o possível local de origem do surto, conforme demonstrado em um surto ocorrido em uma UTI da Alemanha. Tal surto mostrou correlação genética entre os clones identificados nas superfícies contaminadas e os isolados dos pacientes (10).

Este estudo pode apresentar viés de seleção, visto que os casos foram identificados com base nos resultados de cultura de vigilância e diagnósticos para detecção de *Enterococcus* resistentes à vancomicina. De acordo com os registros do hospital, 19 casos com cultura positiva ocorreram no período. Os isolados estavam disponíveis para caracterização microbiológica e molecular em apenas 15 casos.

O surto de *Enterococcus* resistentes à vancomicina enfrentado nas UTI dedicadas a pacientes com covid-19 destacou vulnerabilidades críticas nas práticas de controle de infecção. As intervenções focadas na revisão e reestruturação dos procedimentos de limpeza e desinfecção foram decisivas para a interrupção do surto. A identificação das pias na sala de utilidades como potencial local de cruzamento entre materiais sujos e já desinfetados e a subsequente intervenção para evitar esse contato parecem ter contribuído para o controle dos casos. A identificação de dois perfis clonais entre os isolados reforça a complexidade do controle de infecções e a importância de adaptação dinâmica às mudanças nos padrões de transmissão.

Este estudo reforça a necessidade de controle rigoroso de infecções hospitalares, especialmente durante pandemias, ao apresentar abordagem de investigação e controle de surto de Enterococcus resistente à vancomicina. A contaminação cruzada entre materiais desinfetados e sujos na pia da sala de utilidades/expurgo foi identificada como fator contribuinte. Medidas corretivas, como a organização dos materiais em locais específicos e a garantia de fluxo unidirecional de desinfecção, foram essenciais para o controle do surto, em conformidade com as boas práticas recomendadas pela RDC nº 63/2011 da Agência Nacional de Vigilância Sanitária, que orienta a adoção de procedimentos de limpeza e desinfecção em áreas críticas. Sugere-se o desenvolvimento de manual de boas práticas para áreas de apoio hospitalar, como a sala de utilidades, conforme recomendado também pela RDC nº 50/2002, para prevenir surtos e controlar infecções relacionadas à assistência à saúde.
